# The Korean Speech Recognition Sentences: A Large Corpus for Evaluating Semantic Context and Language Experience in Speech Perception

**DOI:** 10.1044/2023_JSLHR-23-00137

**Published:** 2023-09-06

**Authors:** Jieun Song, Byungjun Kim, Minjeong Kim, Paul Iverson

**Affiliations:** a School of Digital Humanities and Computational Social Sciences, Korea Advanced Institute of Science and Technology, Daejeon, South Korea; b Center for Digital Humanities and Computational Social Sciences, Korea Advanced Institute of Science and Technology, Daejeon, South Korea; c Graduate School of Culture Technology, Korea Advanced Institute of Science and Technology, Daejeon, South Korea; d Department of Speech, Hearing and Phonetic Sciences, University College London, United Kingdom

## Abstract

**Purpose:**

The aim of this study was to develop and validate a large Korean sentence set with varying degrees of semantic predictability that can be used for testing speech recognition and lexical processing.

**Method:**

Sentences differing in the degree of final-word predictability (predictable, neutral, and anomalous) were created with words selected to be suitable for both native and nonnative speakers of Korean. Semantic predictability was evaluated through a series of cloze tests in which native (*n* = 56) and nonnative (*n* = 19) speakers of Korean participated. This study also used a computer language model to evaluate final-word predictabilities; this is a novel approach that the current study adopted to reduce human effort in validating a large number of sentences, which produced results comparable to those of the cloze tests. In a speech recognition task, the sentences were presented to native (*n* = 23) and nonnative (*n* = 21) speakers of Korean in speech-shaped noise at two levels of noise.

**Results:**

The results of the speech-in-noise experiment demonstrated that the intelligibility of the sentences was similar to that of related English corpora. That is, intelligibility was significantly different depending on the semantic condition, and the sentences had the right degree of difficulty for assessing intelligibility differences depending on noise levels and language experience.

**Conclusions:**

This corpus (1,021 sentences in total) adds to the target languages available in speech research and will allow researchers to investigate a range of issues in speech perception in Korean.

**Supplemental Material:**

https://doi.org/10.23641/asha.24045582

Sentence corpora are essential to speech perception research because they involve the phonetic, lexical, semantic integration, and syntactic processes required to understand speech in real-world contexts, but they are short and controllable enough for experimental designs and clinical testing (e.g., Bench et al., 1979; Kalikow et al., 1977). For example, sentences with varying degrees of semantic predictability can be useful for investigating how listeners use semantic–contextual information in a sentence to overcome the difficulty of speech recognition in adverse listening conditions (e.g., Boothroyd & Nittrouer, 1988; Borghini & Hazan, 2020; Kalikow et al., 1977; Obleser et al., 2007; Song et al., 2020; Stringer & Iverson, 2019b). The semantic predictability of the final word can be particularly important in electroencephalography (EEG) experiments with spoken sentences, such as those that evaluate lexical processing using the N400 event-related potential (ERP) component, because the final-word ERP can be measured without overlapping neural potentials from following words (e.g., Federmeier et al., 2007; Song & Iverson, 2018). Such sentences can include highly predictable final words (e.g., *My children enjoy singing simple SONGS*), neutral words with less semantically constraining sentence contexts (e.g., *The students enjoy hearing simple SONGS*), and anomalous words that violate semantic expectations (e.g., *My children enjoy singing simple BOOKS*). Such sentences can also be used for a variety of other purposes, such as investigations of aging, hearing impairment, or second-language (L2) speech perception (e.g., Holmes et al., 2018; Pichora-Fuller, 2008).

The aim of this study was to develop a Korean corpus that varies final-word predictability, similar to typical English test materials that control the predictability of a final noun (e.g., Stringer & Iverson, 2019b). Having similar sentence materials in languages with different linguistic characteristics (e.g., sound inventories, speech rhythm) can allow researchers to make cross-linguistic comparisons in speech research (e.g., Arvaniti, 2012; Ding et al., 2015; Varnet et al., 2017). Moreover, examining differences in speech recognition between the first language (L1) and L2 of bilingual speakers, rather than the more typical between-listener comparisons with a single language (e.g., English perceived by native speakers of English and Korean), can further our understanding of the dual linguistic system of bilingual speakers in which L1 and L2 systems change dynamically and interact with each other (e.g., de Leeuw, 2018; Schmid & Köpke, 2017).

That being said, equivalent sentences can be difficult to create across languages with different linguistic structures. For example, Korean is a head-final language with the subject–object–verb word order. Sentences in Korean typically end with a verb or an adjective (predicate) that takes specific phrases earlier in the sentence as “arguments” that express thematic relations with the predicate (e.g., the agent, “doer” or the theme, “undergoer” of the event described by the verb; Nam, 2007); the type of argument is often marked by case particles (e.g., subject, object, dative). Preceding phrases denoting specific thematic roles thus constrain what could follow at the end as the predicate (e.g., phrases denoting the agent and the goal can be followed by a verb like “go”), thereby limiting possible word choices for the final position without any strong semantic context. We thus do not know whether varying the predictability of the final word will have the same effect in Korean as does varying the final-noun predictability in English, because words in equivalent English sentences (e.g., Stringer & Iverson, 2019b) do not provide as much grammatical information about the final noun.

This new corpus was needed because no such Korean sentence materials are available. One of the largest corpora in Korean is the Korean Hearing in Noise Test sentences (Moon et al., 2005); they consist of 250 sentences that are relatively colloquial and simple (average syllable count per sentence: 9.2). Jang et al. (2008) developed Korean standard sentence lists for adults and school-aged children, which contain eight lists of 10 sentences for each of the groups. Sentences of both Moon et al. (2005) and Jang et al. (2008) were designed to be suitable for speech-in-noise perception tests (e.g., they have even distribution of phonemes across lists), but they do not provide varying conditions of semantic context. There is a Korean version of the Speech Intelligibility in Noise Test (J. S. Kim et al., 2000) that includes high- and low-probability sentences, but the target word was a noun in various locations before the final word in each sentence. Moreover, the intelligibility of the target words did not differ between the two semantic conditions, which appeared to have been caused by methodological issues such as the questions they used to ask the target word (cf. An et al., 2002).

Recently, English sentence materials have been developed specifically for testing nonnative listeners (Calandruccio & Smiljanic, 2012; Stringer & Iverson, 2019b), using words and syntactic structures that are not complex for them to understand. Over the last couple of decades, the number of learners of Korean has increased rapidly (Y. Park, 2015), and so has the number of foreign residents in South Korea, with an annual increase of over 8% in the past 6 years (Statistics Research Institute, 2021). The number of test takers of the Test of Proficiency in Korean (TOPIK) per year increased from 2,274 in 1997 to around 40,000 in 2020 (Korean Institute for Curriculum and Evaluation, 2010; Public Data Portal, n.d.). Testing speech recognition of both native and nonnative speakers of Korean will thus become increasingly important. To make test sentences suitable for foreign-language speakers, careful consideration needs to be made during the sentence development process as regards to vocabulary and sentence complexity; the materials should be designed to measure differences in their speech perception rather than their knowledge of complex vocabulary or grammar.

Our corpus (Korean Speech Recognition [KSR] sentences) differs from the previous Korean sentence materials in three key ways: (a) KSR sentences consist of sentence triplets, which vary in terms of the predictability of final words (predictable, neutral, and anomalous sentences); (b) our sentences were designed to be suitable for testing both native and nonnative adult speakers of Korean in terms of sentence complexity and vocabulary; (c) we developed a large-scale sentence set containing 1,021 sentences in total, which can be particularly useful for experiments that require a great number of trials or multiple experimental conditions. Both content and grammatical words of the sentences were constrained to those that appear in a vocabulary list for learners of Korean. This was done so that speech recognition by nonnative listeners would not be simply impaired by their lack of familiarity of the words. We created a large number of sentences for this corpus, primarily because EEG typically requires averaging across many trials (e.g., 20–100; Šoškić et al., 2021); a smaller sentence set would be sufficient for a simple comparison (e.g., low vs. high predictability), but a larger set allows for more experimental manipulations (e.g., noise level, noise type, accent differences, dual tasks).

This study also adopted a novel approach to developing a sentence corpus with varying degrees of semantic predictability by combining a traditional method of conducting cloze probability tests with a deep learning language model. Specifically, we performed a series of cloze probability tests with native and nonnative speakers of Korean and modified the sentences based on the results of the cloze tests. A deep learning language model was then used to predict the final word of each sentence given the sentence context; the prediction outcomes were used to further evaluate the semantic probabilities of the final words. We took this new approach in an attempt to enhance the validity of the semantic conditions and to reduce human effort in evaluating sentences. Language models have recently demonstrated remarkable performance in various natural language tasks (e.g., Li, 2022), but they have not been previously used in sentence material development for language research.

Ultimately, the aim of developing the KSR corpus was to make Korean sentences available for speech recognition tasks in a variety of research studies, including those examining the effects of semantic context, the listener's language experience, and noise. To evaluate the suitability of the KSR corpus for these purposes, we examined the recognition of the sentences by native and nonnative speakers of Korean in noise. Differences in intelligibility depending on the semantic condition and the listener group are more likely to emerge in noisy conditions. In addition, previous research conducted using materials of other languages has shown that nonnative listeners can have reduced ability to benefit from semantic cues in difficult listening conditions (see Lecumberri et al., 2010, for a review) and that semantic integration can be slower and more effortful in nonnative listeners (e.g., Hahne, 2001; Song et al., 2020). In this study, we examined how the effects of semantic context, language experience (i.e., native vs. nonnative listeners), and noise interact with one another in determining the intelligibility of the KSR sentences in a behavioral speech recognition task.

## Method

### Sentence Development

The development process of the KSR sentences was similar to that of Stringer and Iverson (2019b), as described in detail below. However, one of the main differences between the two sentence sets was the type of the final word. It was always a noun in Stringer and Iverson (2019b), whereas in the KSR sentences, the final word was always either a verb or an adjective, and it contained a past- or present-tense morpheme followed by a sentence-ending morpheme “*–da*” indicating a declarative sentence. Some final words had an honorific morpheme “*–si*” when the subject was someone older or superior (e.g., grandmother). We constructed sentences using Korean words that were included in the vocabulary lists of the TOPIK for elementary and intermediate learners of Korean (J. Kim, 2009, 2010). These basic vocabulary lists contained a total of 4,433 content words and 297 grammatical items; they were chosen based on frequencies of words that appeared in language books focused on spoken usage of Korean, TOPIK tests, and the Sejong corpus, a large collection of Korean texts containing both spoken and written Korean (H. Kim, 2006). The majority of the sentences that we created had simple syntactic structures. Some sentences had a relatively complex structure containing conjunctive suffixes connecting two clauses (e.g., *−ko*,^1^ “and”; *−lyeko*, “in order to”; *–myense*, “while doing”) or prenominal suffixes attached to an embedded verb (e.g., −*n*; *kocangna**n** mwun*, “door that broke”). Nonetheless, these sentences were deemed appropriate for nonnative speakers at an intermediate level of proficiency or higher, as the grammatical items (e.g., particles, endings) used were limited to those included in the lists. These sentences were not necessarily long as the subject and the object can easily be omitted in Korean.

Following the method used in Stringer and Iverson (2019b), we made triplets of sentences; a semantically predictable sentence had a highly constraining sentence frame followed by the final word that was congruent with the context. A semantically neutral sentence had the same final word as its predictable counterpart, which was preceded by a neutral context. An anomalous sentence had the same sentence frame as the predictable counterpart (i.e., highly constraining context) but ended with a word that was not congruent with the context (see Table 1).

**Table 1.** Example of sentence triplets.

Condition	Example
Predictable	어린이에게 안전 교육은 매우 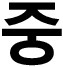 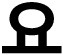 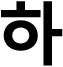 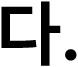 elini-eykey ancen kyoyuk-un maywu cwungyoha-ta child-DAT^a^ safety education-TOP very important-DECL “Safety training is very important for children.”
Neutral	부모와 자식 간에 대화가 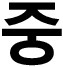 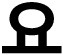 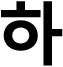 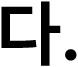 pwumo-wa casik kan-ey tayhwa-ka cwungyoha-ta parent-CONJ child between-DAT conversation-NOM important-DECL “Communication is important between parents and children.”
Anomalous	어린이에게 안전 교육은 매우 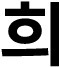 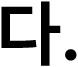 elini-eykey ancen kyoyuk-un maywu huy-ta child-DAT safety education-TOP very white-DECL “Safety training is very white for children.”

a The following abbreviations were used for grammatical items: DAT = dative; TOP = topic; DECL = declarative; CONJ = conjunctive; NOM = nominative.

### Sentence Modification via Cloze Tests

Six cloze tests were performed on the predictable and neutral sentences created (three tests each) to verify the predictability of the final words. In a cloze test, subjects read each sentence with the final word removed and filled out the final word that they thought was most likely to complete the sentence, as shown in Example (1). A word's cloze probability was the percentage of subjects who chose that word to complete the sentence. When their answers contained the stem of the target verb or adjective, but slightly different grammatical morphemes (e.g., different tense markers or an honorific marker –*si*), they were counted as correct answers. We used the results of the cloze tests to modify the sentences further to meet the desired level of final-word cloze probability of each sentence.

(1)  

  상인들이 시장에서 야채를 ____.

  sangin-tul-i sicang-eyse yachay-lul  vendor-PL-NOM market-LOC vegetable-ACC^2^

  “Vendors ____ vegetables at the market.”

Native and nonnative speakers of Korean participated in the cloze tests online via Qualtrics. They were asked to work alone without a dictionary or searching the Internet, and the order of the sentences was randomized. Native subjects were all adult native Korean speakers (age: *M* = 28.9 years; 42 female and 14 male). Nonnative subjects were adults (age: *M* = 24.9 years; 15 female and four male) from various native language backgrounds: seven English, five Indonesian, two Mandarin Chinese, one Cantonese, one Russian, one Arabic, one Dutch, and one Javanese. Their mean length of learning for Korean was 7.4 years (*SD* = 4.5), with the mean onset age of learning of 15.6 years (*SD* = 6.03). They also reported that they had lived in South Korea for about 2.9 years (*SD* = 2.27); they arrived in Korea as adults, except for two subjects who arrived in the country at the age of 17 years. Two subjects reported that they had never lived in Korea and learned Korean in their home country. Their self-reported proficiency levels were “advanced” (10 subjects), “fluent” (six subjects), or “intermediate” (two subjects).^3^ Overall, they were proficient enough in Korean to be able to understand the sentences of the cloze tests and provide a reasonable response, but their length of residence in Korea and onset age of learning suggested that they were late learners without many years of experience in living in Korea. Although the nonnative subjects were smaller in number than the native subjects, most of the nonnative subjects took part in more than one cloze test (one to five tests) so that each cloze test was completed by a similar number of native and nonnative subjects. Tests of the same semantic condition took place 1–3 months apart. It is therefore unlikely that participation in one cloze test affected the results of a subsequent test. Of the native subjects, seven participated in two tests (P1 and N1) that contained completely different sentences, while the remaining subjects only took part in one. The details of each cloze test and the process of sentence modification are described in the following sections.

#### Predictable Sentences

A set of 400 predictable sentences was created first. Ten native speakers and 10 nonnative speakers of Korean participated in the first cloze test (P1). The average final-word cloze probability across all sentences of the initial set was 80.48% for native speakers and 64.60% for nonnative speakers. This indicates that final words of these sentences were highly predictable overall, but nonnative speakers' answers were more varied than those of native speakers. This difference likely occurred because nonnative speakers were less able to choose a word that best fit the context due to their incomplete linguistic knowledge (e.g., collocations, syntax). Following prior research (Block & Baldwin, 2010; Stringer & Iverson, 2019b), sentences were considered sufficiently predictable if they received a final-word cloze probability of 65% or higher. Of 400 sentences that were included in the initial set, 229 passed the inclusion criterion for both native and nonnative speakers, which remained in the predictable sentence set. Four of them received a cloze probability higher than the threshold (i.e., 65%) but with a word different from what was intended in the original sentences, in which case the final word was replaced with the frequent response. One sentence was dropped from this list as its final word had already been used in another sentence in error.

Of the 171 sentences that did not reach the desired level of cloze probability, 75 sentences were modified to strengthen contextual cues based on the answers the participants gave. In most of the sentences, only the words in the sentence frame were changed, while eight sentences had both the final word and the sentence frame modified. The remaining 96 sentences were discarded, and 92 of them were replaced by completely new sentences.

The second cloze test (P2) was then conducted to verify the semantic predictability of the 167 sentences. Thirteen native and 11 nonnative speakers took part in P2. The average final-word cloze probability of the revised set was 85.31% for native speakers and was 66.19% for nonnative speakers. Similar to P1, the lower cloze probability obtained from nonnative speakers left more sentences unqualified for the predictable condition, although some of the sentences were highly predictable for native listeners. It appeared that the language proficiency of nonnative subjects of the current study was not as high as that of nonnative English speakers in the study of Stringer and Iverson (2019b); cloze probabilities were more similar between native and nonnative speakers in their study. The threshold was thus adjusted to a slightly more lenient level, 63.63% for P2. As a result, 102 sentences were added to the final set of predictable sentences.

Nineteen of the remaining 65 sentences were adapted and then verified in the last cloze test for predictable sentences (P3). This test was performed with a smaller group of subjects (five natives, five nonnatives). The average cloze probability was 85.26% and 67.37% for native and nonnative speakers, respectively. Nine sentences received a cloze probability over 65% for both groups of listeners, which were added to the final list of predictable sentences. After the three cloze tests, 340 sentences were retained. The average cloze probability of these sentences was 92.9% for native speakers and 79.3% for nonnative speakers. Table 2 above shows the number of predictable sentences added to the final set after each test.

**Table 2.** Development of predicable sentences.

Cloze test	P1	P2	P3	Total
Number of sentences retained after each cloze test	229	102	9	340

*Note.*  P1 = first cloze test; P2 = second cloze test; P3 = third cloze test.

#### Neutral Sentences

Neutral sentences were made based on 396 predictable sentences that included some of the rejected sentences (56 sentences) after the cloze tests, but they still served as a good basis for creating neutral sentences. A weakly constraining context was created by changing the sentence frame of each predictable sentence. The final word remained unchanged. Because a predicable sentence and its neutral counterpart had the same predicate (i.e., verb or adjective) at the end, they had similar syntactic structures. For example, the Korean verb “chwihata” (“be drunk”) requires two phrases denoting a stimulus and the person who experiences it. Each is realized with specific grammatical case particles in Korean (e.g., −*ey*, −*i*/−*ka*; Nam, 2007), as shown in Example (2). This helped maintain equivalence between the two semantic conditions, although more words were usually added (e.g., adverbial phrases) to both predictable and neutral sentences to create a natural context that was appropriate for each semantic condition.

(2)  

  Predictable sentence

  아빠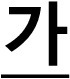 맥주 한잔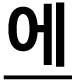 벌써 
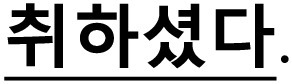

  appa**-ka** maykcwu hancan**-ey** pelsse **chwiha-sy-ess-ta.**father-NOM^4^ beer one glass-by already be drunk-HON-PAST-DECL“Dad was already drunk off a glass of beer.”

  Neutral sentence

  우리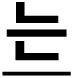 멋진 재즈 연주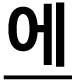 
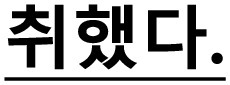

  wuli**-nun** mescin caycu yencwu**-ey chwihay-ss-ta.**

  We-TOC great jazz performance-by be drunk-PAST-DECL

  “We were drawn into the great jazz performance.”

The semantic predictability of the initial set of 396 neutral sentences was tested in a cloze test (N1) with 10 native and 10 nonnative speakers of Korean. Cloze probability was calculated for the word that was reported most frequently in the responses. The mean cloze probability of the sentences in N1 was 42.6% for native speakers and 35.5% for nonnative speakers. Following previous studies (Block & Baldwin, 2010; Stringer & Iverson, 2019b), we used the upper threshold of 40% for the inclusion of neutral sentences. Two hundred one sentences that received a cloze probability under 40% from both groups of listeners were retained. The remaining 194 sentences were adapted to make the sentence context more neutral by taking subjects' responses into consideration.

The second cloze test (N2) was performed to evaluate the predictability of these adapted sentences. Ten native and eight nonnative speakers of Korean took part in N2. The results found that the sentences had an average final-word cloze probability of 33.8% and 32.4% for native and nonnative speakers, respectively. Of the 194 sentences tested, 136 had a cloze probability under 40% for both groups of listeners, and the remaining 58 sentences were excluded from the final set. Meeting the inclusion criterion for neutral sentences (cloze probability under 40%) was more difficult for native listeners than for nonnative listeners. We thus modified 15 sentences that had a cloze probability over 40% only for native speakers and conducted the last cloze test (N3) with 14 native speakers. The results confirmed that all of the modified sentences had a cloze probability under 40% (average: 20.5%). The 15 sentences were thus added to the final set of neutral sentences, resulting in a total of 352 neutral sentences. The final-word cloze probability averaged across all the sentences in the final set was 29.0% for native speakers and 27.8% for nonnative speakers. Table 3 displays the number of neutral sentences that were included in the final set as a result of each test.

**Table 3.** Development of neutral sentences.

Cloze test	N1	N2	N3	Total
Number of sentences retained after each cloze test	201	136	15	352

*Note.* N1 = first cloze test; N2 = second cloze test; N3 = third cloze test.

#### Anomalous Sentences

A semantically anomalous sentence had the same sentence frame as the predictable counterpart, but it was completed by a word that was incongruent with the highly constrained context. For example, the word “suit” does not have semantic properties that are appropriate as the “theme” (“undergoer”) of the event “freeze,” as shown in Example (3) below:

(3)  

Anomalous sentence

회사원들은 주로 정장을 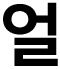

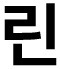

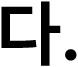

hoysawen-tul-un cwulo cengcang-ul **elli-n-ta**employee of a company-PL^5^-TOP usually suit-ACC freeze-PRES-DECL“Office workers usually freeze a suit.”

Predictable sentence

회사원들은 주로 정장을 
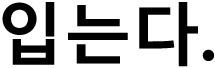

hoysawen-tul-un cwulo cengcang-ul **ip-nun-ta.**employee of a company-PL-TOP usually suit-ACC wear-PRES-DECL“Office workers usually wear a suit.”

In addition, final words in this set were chosen such that they were acoustically distinguishable from the final words of the corresponding predictable sentences with no initial phonological overlap. This consideration is important for studies examining online speech processing (e.g., ERP studies) because the initial phonological overlap can elicit similar responses to the incongruent and congruent words in an early time window (e.g., phonological mapping negativity responses; e.g., Stringer & Iverson, 2019a). In addition, incongruent final words (i.e., verbs or adjectives) were selected so that they did not result in any syntactic violations. That is, despite semantic incongruency, the final words (e.g., freeze) required phrases with the same grammatical items (e.g., object particle “–*ul*” in the above example) as the final words of the predictable counterparts (e.g., wear; see Example [3]). None of the incongruent final words had been used as final words in the other two semantic conditions.

### Further Validation via Language Modeling

In total, there were 340 predictable, 352 neutral, and 396 anomalous sentences after the cloze tests, but one sentence pair (one for each type) was removed because the content of the predictable sentence appeared inappropriate (it contained a gender stereotype). To further validate the final-word predictability of the predictable and neutral sentences, the current study employed a Bidirectional Encoder Representations from Transformers (BERT) language model. BERT achieves state-of-the-art results on a variety of language tasks outperforming other previous language models (Devlin et al., 2019). Its key feature is bidirectional training; it is trained by masking words and predicting the masked words using both the preceding and following contexts. In the current study, the probability of occurrence of the final word was calculated for each sentence using a pretrained Korean BERT model, KLUE RoBERTa large^6^ (S. Park et al., 2021). This model was trained using a large Korean dataset (62 GB) containing about 473 million sentences from various sources, such as news articles, a web-based encyclopedia, and colloquial texts.

We performed a fill-mask task using KLUE RoBERTa large; the final words of the sentences were masked and predicted given the sentential context (e.g., “회사원들은 주로 정장을 <MASK>”; see Example [3] for translation). The task predicted the top 10 words that would replace the masked word with the highest probabilities. The Mean Reciprocal Rank (MRR) was then calculated to compare the predictions of the model with the actual final words of the sentences. MRR is used for evaluating the performance of a model that returns a ranked list of answers (Craswell, 2009); it calculates the reciprocal (1/*N*) of a rank, which gives the value of 1 if the original word is ranked first, 0.5 (1/2) if it is ranked second, and 0.33 (1/3) if it is ranked third. When the model returned multiple forms of the original word with different endings, the higher rank was used to compute the MRR.

The results found that the mean MRR was 0.82 (*SD* = 0.32) for the predictable sentences. In contrast, the mean MRR was much lower for the neutral sentences, with a mean of 0.15 (*SD* = 0.28). That is, the final words of the predictable sentences were usually among the top-ranked words (e.g., first or second) in the model prediction, whereas those of neutral sentences were not, demonstrating highly consistent results between the cloze tests and the model's fill-mask task. The results showed that the MRR was 0 for all the 395 anomalous sentences, confirming that the final words in anomalous sentences were incongruent with the context.

That said, we used the modeling results cautiously, in combination with the results of the cloze tests, for the validation of the corpus. Despite the high consistency between the two, there were a small number of cases in which the model predicted words that were semantically similar to the original word but were not exactly what speakers chose as the most likely candidate (e.g., “*entered*” or “*went [to]*” for “*I <MASK> a company after graduation.*”^7^; they could have the same meaning as the original word “*got a job [at]*” in that context). There were also some neutral sentences whose final words were ranked high relative to other words (thus high MRR values such as 1 or 0.5), despite having low probabilities. The current study thus took into account the results of both the cloze tests and the fill-mask task to find any remaining unsuitable sentences.

Specifically, we found a list of 53 predictable sentences whose MRR scores were lower than 0.5 (i.e., the final word was not the first- or second-ranked word). Among these sentences, we rejected the ones whose final-word cloze probability rated by native or nonnative speakers was lower than 90 (median). In other words, if a sentence received a cloze probability of 90 or higher from both native and nonnative subjects, we concluded that its low MRR value should not be used for rejecting the sentence. Thirty-eight sentences fulfilled the rejection criteria and were therefore removed from the corpus; they had a mean MRR of 0.18. Similarly, we applied this dual verification approach to neutral sentences. Among 48 neutral sentences that received an MRR of 0.5 or higher, we rejected the sentences that had a cloze probability over 30 (median) for native or nonnative subjects; there were 26 sentences like this. That is, the modeling result suggested that these 26 sentences were not suitable as neutral sentences, and their cloze probabilities assessed by human subjects were also not low enough to override the modeling result. We therefore rejected 64 sentences in total using this method (see Table 4). All of the 395 anomalous sentences created were retained in the corpus.

**Table 4.** The number of sentences retained after the model verification by sentence type.

Sentence type	Predictable sentences	Neutral sentences	Total
Number of sentences retained after model verification	301	325	626

### Final Sentence Sets

The properties of predictable and neutral sentences are displayed in Table 5. Final words tended to be longer than those in other English sentence materials (e.g., a syllable count of 1.79 in Stringer & Iverson, 2019b), but it should be noted that the final words (i.e., verbs or adjectives) in this study contained grammatical endings such as a tense marker, which made the final words slightly longer. Predictable and neutral sentences had the same final words, but the syllable counts of the final words were slightly different because they sometimes had different grammatical morphemes attached. As displayed in Table 5, the average number of content words in the sentence frame was slightly greater in the predictable than in the neutral sentences. This may have been because more words were needed to create a highly constrained context. When it is required to keep the number of content words perfectly matched between conditions, researchers can select a subset of sentences that meet the requirement; sentence properties including the syllable count and the number of content words for the full list of sentences are available in Supplemental Material S1.

**Table 5.** Characteristics of the Korean Speech Recognition sentences.

Characteristic	Predictable	Neutral	Anomalous
Syllable count of the entire sentence	13.55 (1.98)	12.78 (1.93)	13.96 (2.06)
Number of content words in the sentence frame	3.37 (0.80)	2.81 (0.67)	3.36 (0.80)
Syllable count of final words	3.17 (0.81)	3.26 (0.79)	3.65 (0.69)

*Note.* Mean values are provided with standard deviation in parentheses.

Predictable and neutral sentences were divided into 12 and 13 lists of approximately 25 sentences, respectively; separate lists were generated for the two semantic conditions. Lists of a sentence material set should have equivalence across them in terms of important characteristics of the sentences (e.g., sentence length, phonetic content), so that intelligibility testing can be reliably conducted using only one of the lists when needed. In previous research, distributing sentences into equivalent lists often required some manual work (e.g., exchanging sentences between lists to achieve a better balance), or the method used for sentence distribution was not reported in detail (e.g., Kalikow et al., 1977; Stringer & Iverson, 2019b). In the current study, the sentences were automatically assigned to each list using the anticlust package (Papenberg & Klau, 2021) in R, such that differences between “clusters” (i.e., lists) were minimized while differences within each cluster were maximized in terms of the following sentence properties: the total number of syllables in the sentence, the number of content words in the sentence frame, the final-word cloze probability, and the number of high-frequency phones (fricatives and affricates) in the sentence. Differences in the mean and standard deviation of these measures were used to find the lists. Researchers can choose any of these balanced lists and use them for their own purposes. If one needs matched pairs of sentences between the semantic conditions (i.e., the same final words for predictable and neutral sentences, the same sentence frame for predictable and anomalous sentences), they can use the 245 complete triplets of sentences available in our corpus.

### Speech-in-Noise Experiment

#### Materials

The predictable and neutral sentences were recorded by four native speakers of Seoul Korean (two female and two male). The audio recordings were made with a RØDE NT1-A microphone in a soundproof booth, with a sample rate of 44100 Hz and a 16-bit quantization rate. Speech-shaped noise was generated for each speaker using a smoothed long-term average spectrum calculated from their sentence recordings, such that masking effects were similar across frequency. The noise was then added to the sentences at a signal-to-noise ratio (SNR) of −2 dB and +3 dB. The SNR of −2 dB was determined after pilot testing to achieve a noise level at which native listeners could recognize a large proportion of words and benefit from contextual cues, but their performance would not be at ceiling (e.g., the same SNR level was chosen in Bradlow & Alexander, 2007). The lower noise level, +3 dB, was also used because pilot testing showed that the performance of nonnative speakers was too poor at −2 dB; their recognition would show clearer differences depending on the semantic condition at a higher SNR.

#### Subjects and Procedure

Twenty-three adult native speakers and 21 adult nonnative speakers of Korean (age: *M* = 23 years, range: 18–32; 22 female and 22 male) took part in this speech-in-nose experiment via the online platform Gorilla (https://gorilla.sc). They self-reported normal hearing and no history of language or neurological disorders. They were paid for their participation. The nonnative speakers were recruited through Korean language courses and an international student association at the Korea Advanced Institute of Science and Technology. They were from various native language backgrounds (five Mandarin, five Indonesian, two Vietnamese, two Mongolian, two English, one Russian, one Kazakh, one Thai, one Urdu, one Czech), but we recruited nonnative subjects who were similar in terms of age of onset of acquisition and length of residence in Korea; all of them were late learners of Korean, with a mean onset age of 19.5 years (*SD* = 3.4). They were living in South Korea at the time of testing except for one subject, and they had lived in South Korea for about 21 months as an adult (*M* = 21.2 months, *SD* = 18.8). Ten of them reported being advanced learners of Korean, 10 reported being intermediate learners, and one reported being a fluent speaker.

The subjects were asked to listen to the sentences over headphones at a comfortable level in a quiet environment and were given practice trials at the beginning to familiarize themselves with the task. After hearing each sentence once, subjects were instructed to type what they heard in the response box. Because they had to listen to a total of 626 sentences, the whole experiment was divided into four sets. Within each set, short breaks were given after each block containing about 50 sentences. Subjects were asked to complete the four sets within 2 days. The sentence assignment to the four speakers was counterbalanced between subjects. Half of the stimuli were presented with noise at +3 dB, and the other half were at −2 dB. The assignment of the sentences to the two noise levels was also counterbalanced between subjects. The order of stimuli was randomized for each subject. The number of content words that were identified correctly was scored manually by one of the authors for each sentence. Answers containing the target content word with different grammatical endings (e.g., tense markers or an honorific marker –*si*) or particles were counted as correct.

## Results

**Figure 1.**
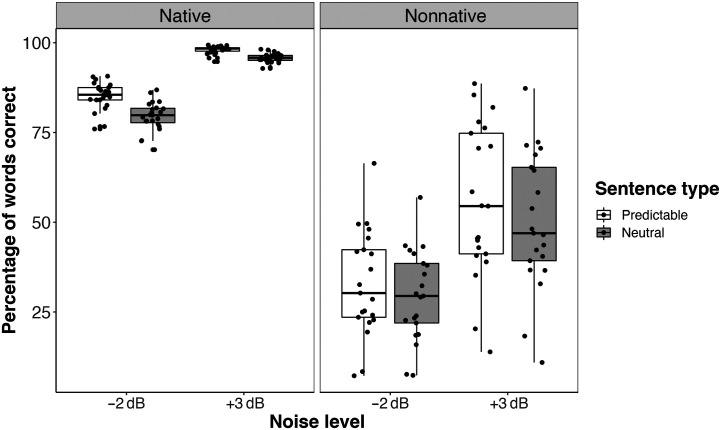
Boxplots showing the percentage of correctly identified words by sentence type, noise level, and listener group. Each dot represents an average percentage score for each individual subject.

As displayed in Figure 1, the accuracy of speech recognition was higher for predictable sentences than for neutral sentences, demonstrating that an English-language approach to constructing sentences that vary in final-word predictability can be applicable to Korean, despite the differences in sentence structure. As expected, speech recognition accuracy was also higher for the +3 dB SNR condition than for the −2 dB condition and for native than nonnative listeners. In addition, the nonnative listeners varied more in their recognition accuracy than did the native listeners. The mean and standard deviation for each condition of sentence type, noise level, and listener group are listed in Table 6. A linear mixed-effects model analysis was performed using the lme4 package (Bates et al., 2015) in R, with the percentage of words that were correctly identified as the dependent variable and sentence type (predictable vs. neutral sentences), noise level (−2 dB vs. +3 dB), and listener group (native vs. nonnative) as fixed effects. We also included the number of content words in the sentence frame as a fixed factor to examine whether or not this difference in predictable and neutral sentences could have any effect on intelligibility. By-subject and by-sentence intercepts were also included in the model. Significance of each factor was calculated by comparing models with and without the relevant factor (i.e., model comparison).

**Table 6.** Intelligibility scores (percentage of words correct) of the Korean Speech Recognition sentences depending on sentence type, noise level (SNR), and listener group.

Noise level	Native listeners		Nonnative listeners	
	Predictable	Neutral	Predictable	Neutral
−2 dB	85.18 (28.42)	79.37 (29.00)	33.08 (34.01)	29.40 (30.42)
+3 dB	98.02 (9.94)	95.74 (12.89)	55.15 (36.66)	50.41 (34.94)

*Note.* Mean values are provided with standard deviation in parentheses. SNR signal-to-noise ratio.

The results demonstrated that the main effect of sentence type was significant, χ^2^(1) = 26.72, *p* < .001, verifying that contextual cues in the predictable sentences helped listeners understand the sentences better. The main effect of listener group was also significant, χ^2^(1) = 74.47, *p* < .001, confirming that native speakers were significantly better at this task than nonnative listeners. As expected, the main effect of noise level was also significant, χ^2^(1) = 2880.6, *p* < .001; the recognition accuracy was significantly higher at the SNR of +3 dB than of −2 dB. We also found a significant two-way interaction of listener group and noise level, χ^2^(1) = 157.06, *p* < .001; this suggests that nonnative speakers were more adversely affected by the increase in the noise level than were native speakers. The two-way interaction of sentence type and listener group was not significant, *p* = .6. That is, both native and nonnative listeners were able to benefit from semantic cues, with similar effects of sentence type. The two-way interaction of sentence type and noise was also not significant, *p* = .79, suggesting that the effect of sentence type was not different between the two noise levels. However, there was a significant three-way interaction of listener group, sentence type, and noise level, χ^2^(3) = 19.91, *p* < .001. This was caused by native listeners' performance being at ceiling at +3 dB, thereby having smaller differences between predictable and neutral sentences than at −2 dB. The effect of the number of content words was not significant, *p* = .82, verifying that this small difference between predictable and neutral sentences is not problematic for measuring intelligibility.

Mixed-effects model analyses were also performed separately for predictable and neutral sentences to examine the effect of list, with the percentage of correctly identified words as the dependent variable and list, listener group, and noise level as fixed effects. By-subject and by-sentence intercepts were included in the models. The main effect of list was not significant for both predictable and neutral sentences with *p* values of .43 and .99, respectively. However, for predictable sentences, there was a significant interaction of list and noise level, χ^2^(22) = 38.14, *p* = .02. A post hoc test was conducted with the multcomp package (Hothorn et al., 2008), which found that at the −2 dB SNR, the intelligibility of List A8 (“A” indicates predictable sentences) was significantly higher than that of List A12 (β = 9.18, *SE* = 3.17, *z* = 2.90, *p* = .04). The difference in intelligibility between List A8 and List A3 and between List A8 and List 9 did not reach significance with a *p* value of .07. However, these differences were not found at the lower noise level (i.e., +3 dB SNR). Similarly, there was a significant two-way interaction of list and listener group, χ^2^(22) = 37.45, *p* = .02. A post hoc test found that List A8 was significantly more intelligible than List A4 for nonnative listeners (β = −9.48, *SE* = 3.11, *z* = −3.04, *p* = .02) and that List A10 was also significantly more intelligible than List A4 for nonnative listeners (β = −9.80, *SE* = 3.11, *z* = −3.15, *p* = .02). In contrast, no differences between the lists were found for native listeners.

**Figure 2.**
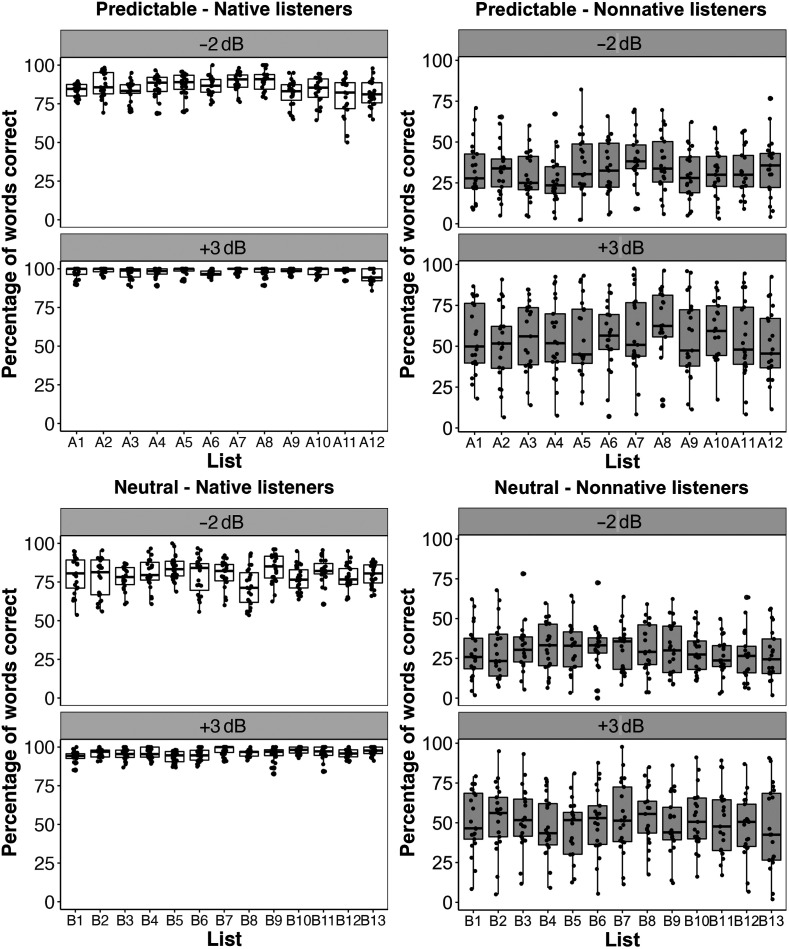
Boxplots showing the percentage of correctly identified words by list and noise level divided into different sentence types (predictable vs. neutral sentences) and listener groups (native vs. nonnative listeners). Each dot represents an average percentage score for each individual subject.

Similarly, there was a significant two-way interaction of list and noise level for neutral sentences, χ^2^(24) = 48.76, *p* = 0.002. A post hoc test revealed that the interaction was caused because List B5 (“B” indicates neutral sentences) received significantly higher intelligibility scores than List B8, but only at −2 dB (β = 9.19, *SE* = 3.01, *z* = 3.05, *p* = .02). In addition, there was a significant interaction of list and listener group, χ^2^(24) = 56.64, *p* < .001. Specifically, List B13 was less intelligible than List B3 for nonnative listeners, but the *p* value was slightly greater than .05 (*p* = .0595). No such differences were found for native listeners. These findings suggest that the 25 lists are overall similar in intelligibility, but sentences of some lists tend to be slightly easier (e.g., List A8, List B5) or more difficult (e.g., List A4, List B8), and these differences could emerge in more difficult conditions (see Figure 2).

## Discussion and Conclusions

This study developed a large-scale sentence material set in Korean (KSR sentences), which can be used for testing speech recognition performance in a variety of speech and language studies. The results of the speech-in-noise experiment showed that the intelligibility of the KSR sentences was similar to that reported in studies that used English sentences with similar noise levels (e.g., Bradlow & Alexander, 2007; Stringer & Iverson, 2019b). Moreover, the KSR sentences have different levels of final-word predictability (i.e., predictable, neutral, and anomalous), which is a feature that is not offered by other Korean sentence materials. The results of the speech-in-noise experiment confirmed that predictable sentences were more intelligible than neutral sentences in noise because of the availability of stronger semantic cues. This demonstrated that Korean sentences, constructed similarly to English materials with varying degrees of final-word predictability, can be used to examine semantically related differences during sentence recognition. In addition, Korean–English bilinguals' speech recognition can be assessed in both of the languages using the KSR sentences in parallel with the Non-Native Speech Recognition sentences (Stringer & Iverson, 2019b).

For sentence development, the current study devised a novel method; in addition to conducting the cloze tests in which subjects were asked to fill out the most plausible final word for each sentence, we used the language model KLUE RoBERTa (S. Park et al., 2021), which predicted words that were most likely to occur given each sentence context. We were able to validate the sentences more thoroughly using this method; we might have otherwise needed more testing with a larger number of subjects to be able to find those 64 sentences that were less suitable. This adds a significant methodological contribution to sentence development research; language models could be used to reduce the human effort required to create and validate a large number of sentences and to increase its validity. For example, it was sometimes difficult to come up with neutral sentences because some words (e.g., a noun phrase followed by a specific verb) went together more naturally than others, although they did not form a strongly constraining context. Because language models can predict such co-occurrence patterns, it will be possible to use them for selecting combinations of words or phrases with varying levels of co-occurrence probabilities when developing sentence corpora. It may also be possible to use generative pretrained transformer models (e.g., ChatGPT; OpenAI, 2022) to reduce the effort in creating sentences (e.g., ones with a specific syntactic structure) from scratch.

In addition, this corpus was designed to be suitable for both native and nonnative speakers of Korean in terms of vocabulary and grammar. In the speech-in-noise experiment, we tested late learners of Korean who had not had many years of experience living in Korea; most of them started learning Korean as an adult (age: *M* = 19.7 years), and their mean length of residence was shorter than 2 years. Compared to native listeners, their performance was overall poor in noise and differed more widely among them, but they were able to exploit contextual cues during sentence recognition; we found similar effects of sentence type for both listener groups (e.g., Borghini & Hazan, 2020), although they can show differences in EEG experiments that examine lexical processing (Song et al., 2020; Stringer & Iverson, 2019a). With regard to the suitability of the KSR corpus as a test set for nonnative speakers, these findings indicate that the KSR sentences have an appropriate level of difficulty for nonnative speakers of Korean including late learners and that semantic cues available in predictable sentences are strong enough to be exploited by them.

It is hoped that our new sentence materials will be used in various psycholinguistic studies examining sentence processing in Korean. The large number of sentences can be used for studies with multiple experimental conditions or those needing a large number of trials within each condition (e.g., ERP studies). One example that requires both varying conditions of semantic predictability and a large number of sentences is ERP studies that measure the N400 response. N400 is known to be larger for words that are less predictable from the preceding context (e.g., see Kutas & Federmeier, 2000, for a review) and can be measured reliably by averaging across multiple trials within each condition (e.g., 30 trials). We have previously used English sentences of Stringer and Iverson (2019b) to measure N400 (Song & Iverson, 2018; Song et al., 2020; Stringer & Iverson, 2019a), and we hope that the current sentence set will likewise be used for similar experiments. Anomalous sentences can also be used as catch trials for ensuring that subjects maintain their attention on stimuli (e.g., Song & Iverson, 2018) and for investigating effects of violation of real-world knowledge or animacy on speech comprehension (e.g., Vega-Mendoza et al., 2021).

Moreover, our sentence materials will help diversify target languages in nonnative speech research. More researchers have begun to investigate speech perception by nonnative speakers of Korean, but the research thus far has mostly focused on the perception of specific Korean phonetic contrasts (e.g., Korean stops; Ahn et al., 2017; Holliday, 2018). The use of our materials can expand the scope of the research to speech comprehension and processing and can open up more questions about nonnative speech perception using a target language with different linguistic characteristics. For example, Korean is an agglutinative language in which affixes are attached to the root of a word to have a variety of grammatical functions (e.g., past, passive, honorific). More research can be carried out in the future using Korean sentences to investigate cross-linguistic differences in speech processing caused by differences in morphosyntax (e.g., Bañón & Marin, 2021). Furthermore, our sentences can be used for listening proficiency tests for learners of Korean in practical settings (e.g., language centers) and in applied linguistics research.

While the development process resembled that of Stringer and Iverson (2019b), different linguistic considerations had to be made for developing equivalent sentences in Korean, and there were some challenges we faced. For example, depending on what syntactic elements (e.g., object) the predicate (i.e., a verb or an adjective at the end) needs, words in the sentence frame had to have the same grammatical endings (e.g., case particles marking subject, object, or location) attached in predictable sentences and neutral counterparts. This restriction made it more difficult to create appropriate contextual cues for each sentence type. It was also difficult to create sentences that were sufficiently predictable for nonnative speakers. High-proficiency learners of Korean are often difficult to find for testing than those of English; nonnative subjects of our cloze tests were less proficient in Korean than those of Stringer and Iverson (2019b) were in English and were thus less familiar with co-occurrence of words in sentences. Despite these challenges, sentences with varying levels of final-word predictability were successfully developed in the current study.

More studies may be required to further evaluate the validity of our materials for testing different listener populations (e.g., children or hearing-impaired listeners), especially in order to use them in clinical environments. Researchers can also freely modify the sentences depending on specific questions or experimental conditions of their research. The current materials will pave the way for a range of new investigations in speech research using the Korean language.

## Data Availability Statement

The sentences developed in this study and their properties are included in this published article and Supplemental Material S1. Other datasets generated or analyzed during the current study are available from the corresponding author on reasonable request.

## Supplementary Material

10.1044/2023_JSLHR-23-00137SMS1 Supplemental Material S1 Full list of sentences.

## Footnotes

1 The Yale system was used for romanizations of Korean.

2 PL = plural; NOM = nominative; LOC = locative; ACC = accusative.

3 One subject (native language: English) informally provided us with information about his Korean language experience but omitted to fill out the questionnaire. He stated that he was a high-proficiency learner and had studied Korean at a university for 4 years.

4 NOM = nominative; HON = honorific; PAST = past tense; DECL = declarative; TOC = topic.

5 PL = plural; TOP = topic; ACC = accusative; PRES = present tense; DECL = declarative.

6
https://huggingface.co/klue/roberta-large.

7 나는 졸업 후에 회사에 들어갔다/갔다/취직했다; na-nun colep hwu-ey hoysa-ey tuleka/ka/chwicikhay-ss-ta; I-TOP graduation after-LOC company-LOC enter/go/get a job-PAST-DECL.
